# Deep Convolutional Neural Networks Outperform Feature-Based But Not Categorical Models in Explaining Object Similarity Judgments

**DOI:** 10.3389/fpsyg.2017.01726

**Published:** 2017-10-09

**Authors:** Kamila M. Jozwik, Nikolaus Kriegeskorte, Katherine R. Storrs, Marieke Mur

**Affiliations:** ^1^Neural Dynamics of Visual Cognition, Department of Education and Psychology, Free University of Berlin, Berlin, Germany; ^2^Memory and Perception Group, MRC Cognition and Brain Sciences Unit, University of Cambridge, Cambridge, United Kingdom

**Keywords:** object recognition, similarity judgments, deep neural networks, categories, features, weighted representational modeling, representational similarity analysis

## Abstract

Recent advances in Deep convolutional Neural Networks (DNNs) have enabled unprecedentedly accurate computational models of brain representations, and present an exciting opportunity to model diverse cognitive functions. State-of-the-art DNNs achieve human-level performance on object categorisation, but it is unclear how well they capture human behavior on complex cognitive tasks. Recent reports suggest that DNNs can explain significant variance in one such task, judging object similarity. Here, we extend these findings by replicating them for a rich set of object images, comparing performance across layers within two DNNs of different depths, and examining how the DNNs’ performance compares to that of non-computational “conceptual” models. Human observers performed similarity judgments for a set of 92 images of real-world objects. Representations of the same images were obtained in each of the layers of two DNNs of different depths (8-layer AlexNet and 16-layer VGG-16). To create conceptual models, other human observers generated visual-feature labels (e.g., “eye”) and category labels (e.g., “animal”) for the same image set. Feature labels were divided into parts, colors, textures and contours, while category labels were divided into subordinate, basic, and superordinate categories. We fitted models derived from the features, categories, and from each layer of each DNN to the similarity judgments, using representational similarity analysis to evaluate model performance. In both DNNs, similarity within the last layer explains most of the explainable variance in human similarity judgments. The last layer outperforms almost all feature-based models. Late and mid-level layers outperform some but not all feature-based models. Importantly, categorical models predict similarity judgments significantly better than any DNN layer. Our results provide further evidence for commonalities between DNNs and brain representations. Models derived from visual features other than object parts perform relatively poorly, perhaps because DNNs more comprehensively capture the colors, textures and contours which matter to human object perception. However, categorical models outperform DNNs, suggesting that further work may be needed to bring high-level semantic representations in DNNs closer to those extracted by humans. Modern DNNs explain similarity judgments remarkably well considering they were not trained on this task, and are promising models for many aspects of human cognition.

## Introduction

Deep convolutional Neural Networks (DNNs) have revolutionized computer vision in recent years, reaching human-level performance on a variety of tasks, including the classification of objects in images (Krizhevsky et al., 2012; Simonyan and Zisserman, 2015; He et al., 2016). DNNs are successful because they can learn efficient representations of rich inputs, such as colored real-world object images. The human brain is faced with the same challenge of learning efficient representations of rich sensory inputs, in order to successfully interact with the world. Neuroscientists have started using DNNs to predict brain representations of images in humans and non-human primates. Results are promising: DNNs predict neural representations of object images as measured in humans via fMRI (Khaligh-Razavi and Kriegeskorte, 2014; Güçlü and van Gerven, 2015) and MEG (Cichy et al., 2016), and in monkeys via electrophysiology (Yamins et al., 2014; Hong et al., 2016). These findings suggest that there are considerable similarities between DNN and brain representations of visual inputs.

Deep convolutional Neural Networks also present an exciting opportunity to model cognitive function. Representations in high-level visual cortex have been shown to predict human similarity judgments well (Haushofer et al., 2008; Op de Beeck et al., 2008; Drucker and Aguirre, 2009; Mur et al., 2013). As representations are similar between high-level visual cortex and late layers of DNNs (Khaligh-Razavi and Kriegeskorte, 2014; Güçlü and van Gerven, 2015), deep nets might be able to predict behavior in cognitive tasks based on these representations, such as judging object similarity. However, even DNNs capable of near-human-level object classification performance classify certain images in highly counterintuitive ways, bringing into question their commonalities with human perception (Szegedy et al., 2013; Nguyen et al., 2014). Neuroscientists have started exploring the suitability of DNNs for modeling behavior in cognitive tasks. DNNs have proven successful in predicting judgments of category typicality (Lake et al., 2015), object memorability (Dubey et al., 2015), and shape sensitivity for abstract shapes, letters, and line drawings and grayscale photographs of real-world objects (Kubilius et al., 2016). Recently, Peterson et al. (2016) demonstrated that, after reweighting features within the network, similarity of activity patterns within a DNN explained the majority of variance in human similarity judgments of animal images. One of the questions we explore here is whether these results extend to real-world object images from a wide range of categories, including animate as well as inanimate objects.

Importantly, DNNs did not fully explain object similarity judgments in Kubilius et al. (2016). It is unclear which aspects of the representation contribute to the explanatory power of DNNs, and which aspects might be missing. Furthermore, it is not known how DNN performance compares to that of simpler conceptual models. DNNs are complex computational models with many parameters and it may not be intuitive what stimulus information is represented at each of the DNN layers. We might gain a better understanding of the nature of DNN representations by comparing the performance of DNNs to that of conceptual models derived from human perception.

Here, we determine how well object representations within each layer of two DNNs with different depths, i.e., 8-layer AlexNet (Krizhevsky et al., 2012) and 16-layer VGG-16 (Simonyan and Zisserman, 2015), predict human object-similarity judgments for a set of 92 colored real-world object images from a wide range of categories (Mur et al., 2013). We compare the performance of DNNs to the performance of conceptual models derived from human perception, using representational similarity analysis (RSA; Kriegeskorte et al., 2008). Our conceptual models are based on visual-feature and category descriptions of the 92 objects, generated by human observers (Jozwik et al., 2016). We show that the last DNN layer explains similarity judgments to a considerable extent, and performs better than earlier layers. Reweighting model units (fully-connected layers) or feature maps (convolutional layers) improves performance for images held out during fitting for most fully-connected layers, and for late, but not intermediate and early, convolutional layers. Performance of late convolutional layers remains significantly below the performance of the last layer. Importantly, we show that the last DNN layer outperforms most feature-based, but not categorical models at explaining human similarity judgments.

## Materials and Methods

### Object-Similarity Judgments

Acquisition and analysis of the object-similarity judgments have been described in Mur et al. (2013), where further details can be found.

#### Participants

Sixteen healthy human volunteers participated in the similarity-judgment experiment (mean age = 28 years; 12 females). Participants had normal or corrected-to-normal vision; 13 of them were right-handed. Before completing the experiment, participants received information about the procedure of the experiment and gave their written informed consent. The experiment was conducted in accordance with the Ethics Committee of the Faculty of Psychology and Neuroscience, Maastricht University, Netherlands.

#### Stimuli

**FIGURE 1:**
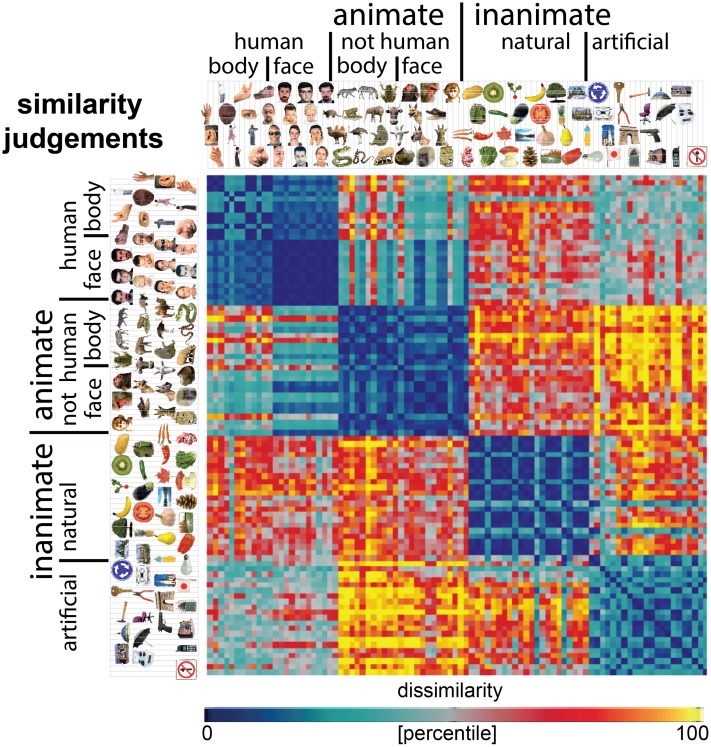
Similarity-judgment RDM. Representational dissimilarity matrix (RDM) showing object-similarity judgments for a set of 92 colored object images. The RDM is based on similarity judgments from 16 human participants, averaged at the level of the dissimilarities. Each entry of the RDM represents the judged dissimilarity between two images. RDM values were transformed into percentiles for visualization (see color bar). The similarity judgments show four main clusters corresponding to humans, non-human animals, natural objects, and manmade objects, and a tight cluster of human faces.

Stimuli were 92 colored images of real-world objects spanning a range of categories, including humans, non-human animals, natural objects, and artificial objects (**Figure 1**). Objects were segmented from their backgrounds and presented on a gray background.

#### Experimental Design and Task

We acquired pairwise object-similarity judgments for all 92 object images by asking participants to perform a multi-arrangement task (Kriegeskorte and Mur, 2012). During this task, the object images are shown on a computer screen in a circular arena, and participants are asked to arrange the objects by their similarity, such that similar objects are placed close together and dissimilar objects are placed further apart. The multi-arrangement method uses an adaptive trial design, showing all object images on the first trial, and selecting subsets of objects with weak dissimilarity evidence for subsequent trials. This method allows efficient acquisition of a large number of pairwise similarities. Participants performed the task for 1 h, during which they completed 22 trials on average (standard deviation across participants = 11.4). Excluding the first trial, the number of objects per trial was 16 on average (first averaged across trials, then across participants; standard deviation across participants = 3.4). We deliberately did not specify which object properties to focus on, to avoid biasing participants’ spontaneous mental representation of the similarities between objects. Our aim was to obtain similarity judgments that reflect the natural representation of objects without forcing participants to rely on one given dimension. However, participants were asked after having performed the task, what dimension(s) they used in judging object similarity. All but one of the 16 participants reported arranging the images according to a categorical structure. The categories mentioned by the participants correspond to the (sub)clusters shown in **Figure 1**, including human faces, monkeys/apes, fruits, and tools. Most participants indicated that, within category clusters, they used shape and color to arrange the objects. The reports suggest that participants used a consistent strategy throughout the experiment. Consistent with this observation, similarity judgments were highly positively correlated between each new trial and the average of the previous trials (Pearson’s *r* = 0.98; first averaged across trials, then subjects, using Fisher z transformation).

#### Construction of the Behavioral Representational Dissimilarity Matrix

Participants were instructed to use the entire arena for arranging the object images on each trial. Consequently, only the relations between distances on a single trial, not the absolute on-screen distances, were meaningful. For each participant, dissimilarity estimates (on-screen distances between each image pair) were therefore averaged across trials using an iterative procedure, alternately scaling the single-trial estimates to match their weighted average, and recomputing the weighted average, until convergence (Kriegeskorte and Mur, 2012). This resulted in an estimate of the dissimilarity between every pair of stimuli, and these values were placed in a representational dissimilarity matrix (RDM). RDMs were constructed for each participant separately and then combined by averaging across participants. The resulting RDM (**Figure 1**) captures the cognitive similarity between the objects in our set, with some stimulus attributes emphasized and others de-emphasized in object perception.

### Deep Neural Network Representations

#### Architectures of Deep Neural Networks

Representations of the same set of 92 images were computed from the layers of two convolutional neural network architectures with different depths. The first DNN was AlexNet (Krizhevsky et al., 2012) with eight layers. The second DNN was VGG-16 (Simonyan and Zisserman, 2015) with 16 layers. We chose these DNNs because they were the best-performing models in the ImageNet Large Scale Visual Recognition Challenge (Russakovsky et al., 2015) in 2012 and 2014, respectively; and their architectures are relatively simple as compared to other DNNs. In that competition, the VGG network achieved 7.4% top-5 error rate, whereas AlexNet achieved 15.4% top-5 error rate. For reference, Microsoft’s 150-layer network recently obtained 4.94% top-5 error rate outperforming non-expert humans at 5.1% (He et al., 2016). AlexNet and VGG-16 were trained on 1.2 million images from the ImageNet database. The task was to classify each image as containing an object in one of 1,000 possible categories.

The DNN architectures share several principles with the architecture of the primate visual system, in particular: hierarchical organization, convolution, and pooling (Kriegeskorte, 2015; Yamins and DiCarlo, 2016). The hierarchical series of layers transforming information from simple features to a categorical representation mimics the successive cortical regions in the primate visual system. Convolution is inspired by biological vision where local features are replicated across the visual field. Pooling allows tolerance to position of image features, echoing the increasing view tolerance along the primate ventral stream (see Kriegeskorte, 2015; Yamins and DiCarlo, 2016 for further discussion).

**FIGURE 2:**
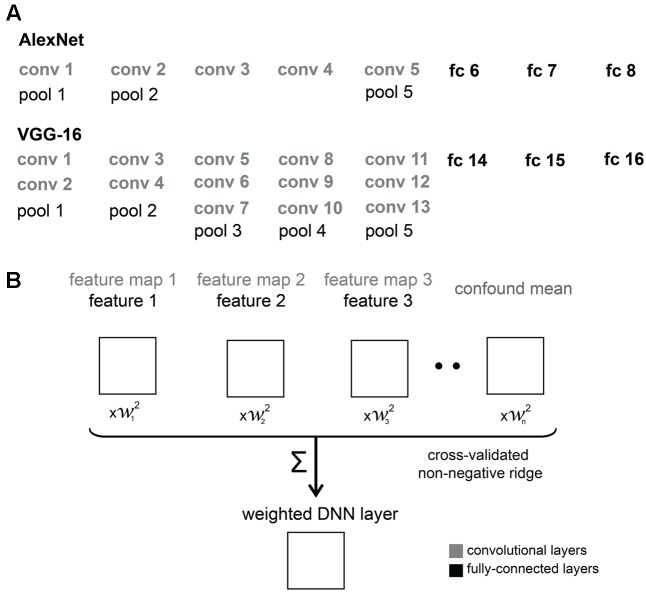
DNN architectures and feature weighting. **(A)** Comparison of AlexNet and VGG-16 architectures. We used convolutional (conv) and fully-connected (fc) layers from AlexNet and VGG-16 in our analyses. **(B)** Schematic overview of feature weighting. The schematic shows a set of example RDMs characterizing the stimulus information represented by the DNNs. For convolutional layers, we created RDMs from activations of feature maps. For fully-connected layers, we created RDMs from activations of individual features, i.e., model units. Within each DNN layer, we used regularized (non-negative ridge) linear regression to estimate the RDM weights that best predict the similarity-judgment RDM. Each DNN layer includes a confound-mean predictor (intercept). The weights were estimated using a cross-validation procedure to prevent overfitting to a particular set of images.

The architectures of the two DNNs are schematically represented in **Figure 2**. AlexNet consists of eight layers, comprising five convolutional and three fully-connected layers. Three max-pooling layers follow the first, second and fifth convolutional layers. Each convolutional layer contains a number of “feature maps”, each of which consists of a single learned filter, applied systematically across spatial locations in the input layer (i.e., convolved with the input). The first convolutional layer has 96 feature maps, the second convolutional layer has 256, and the third, fourth and fifth convolutional layers have 384, 384 and 256 feature maps, respectively. VGG-16 consists of 16 layers including 13 convolutional and three fully-connected layers. Convolutional layers form five groups and each group is followed by a max-pooling layer. The number of feature maps increases from 64, through 128 and 256 until 512 in the last convolutional layers. Within each feature map, the size of the convolutional filter is analogous to the receptive field of a neuron. Units in VGG-16 have smaller receptive fields than in AlexNet across early layers, but both DNNs have the same size filters in the last pooling layer. We used convolutional (conv) and fully-connected (fc) layers from both networks in our analyses.

#### Construction of the DNN Representational Dissimilarity Matrices

For each convolutional layer of each DNN, we extracted the activations in each feature map for each image, and converted these into one activation vector per feature map. Then, for each pair of images we computed the dissimilarity (squared Euclidean distance) between the activation vectors. This yielded a 92 × 92 RDM for each feature map of each convolutional DNN layer. For each fully-connected layer of each DNN, we extracted the activation of each single model unit for each image. For each pair of images, we computed the dissimilarity between the activations (squared Euclidean distance; equivalent here to the squared difference between the two activations). This yielded a 92 × 92 RDM for each model unit of each fully-connected DNN layer. The feature-map and model-unit RDMs capture which stimulus information is emphasized and which is de-emphasized by the DNNs at different stages of processing.

### Conceptual Models

We created conceptual models from object labels generated by human observers. Further details on the acquisition of the object labels can be found in Jozwik et al. (2016).

#### Definition of Conceptual Models

To create conceptual models, human observers generated visual-feature labels (e.g., “eye”) and category labels (e.g., “animal”) for the 92 images. Feature labels were divided into parts, colors, textures and contours, while category labels were divided into subordinate categories, basic categories and superordinate categories. Labels were obtained in a set of two experiments. In Experiment 1, a group of 15 human observers generated visual-feature and category labels for the object images. In the instruction, we defined visual features as “visible elements of the shown object, including object parts, colors, textures and contours”. We defined a category as “a group of objects that the shown object is an example of”. Observers generated 212 visual-feature labels and 197 category labels. These labels are the model dimensions. In Experiment 2, a separate group of 14 human observers judged the applicability of each model dimension to each image, thereby validating the dimensions generated in Experiment 1, and providing, for each image, its value (present or absent) on each of the dimensions. The image values on the validated model dimensions define the model. To increase the stability of the models during subsequent fitting, highly-correlated model dimensions were merged by averaging the image values across these dimensions. The final full feature-based and categorical models consisted of 120 and 114 dimensions, respectively. Model dimensions are listed in Supplementary Table 1.

#### Construction of the Conceptual Representational Dissimilarity Matrices

For each model dimension, we computed, for each pair of images, the squared difference between their values on that dimension. The squared difference reflects the dissimilarity between the two images in a pair. Given that a specific visual feature or category can either be present or absent in a particular image, image dissimilarities along a single model dimension are binary: they are zero if a visual feature or category is present or absent in both images, and one if a visual feature or category is present in one image, but absent in the other. The dissimilarities were stored in an RDM, yielding as many RDMs as model dimensions. The full feature-based RDM model consisted of 120 RDMs; the full categorical RDM model consisted of 114 RDMs. We also included RDM models for subsets of visual features and categories. Visual features were divided into object parts (e.g., “eye”, “arm”), colors (e.g., “red”, “green”), textures (e.g., “stubbly”, “plastic”), and contours (e.g., “curved”, “rectangular”). Categories were divided into subordinate categories (e.g., “great dane”, “roundabout sign”), basic categories (e.g., “face”, “male”), and superordinate categories (e.g., “artificial”, and “organism/living”).

### Weighted Representational Modeling

To improve model performance, we weighted the different model dimensions (feature maps for the convolutional DNN layers; model units for the fully-connected DNN layers; visual features and categories for the conceptual models) to yield an object representation that best predicts the similarity judgments. We used the squared Euclidean distance as our RDM measure. The squared distances sum across model dimensions, so the unfitted model prediction is the sum of the single-dimension RDMs. We estimated the representational weights, one for each single-dimension RDM, using regularized (L2) linear regression, implemented in Matlab using glmnet (Qian et al., 2013). Glmnet implements elastic net regularized regression using cyclical coordinate descent. We used standard settings (including standardization of the predictors before fitting), except that we constrained the weights to be non-negative. To prevent biased model performance estimates due to overfitting to a particular set of images, model performance was estimated by cross-validation to a subset of the images held out during model fitting. For each cross-validation fold, we randomly selected 84 of the 92 images as the training set, and used the corresponding pairwise dissimilarities to estimate the model weights. The model weights were then used to predict the pairwise dissimilarities for the eight left-out images. This procedure was repeated until predictions were obtained for all pairwise dissimilarities. For each cross-validation fold, we determined the best regularization parameter (i.e., the one with the minimum squared error between prediction and data) using nested cross-validation to held-out images within the training set.

### Inferential Analysis on Model Performance

We used the RSA toolbox for inferential analyses (Nili et al., 2014). We quantified model performance by measuring the correlation between the similarity judgments and the dissimilarities predicted by the DNNs and conceptual models. We estimated the correlation using Kendall’s rank correlation coefficient tau a. Given that some of our categorical models predict tied ranks, Kendall’s tau a is an appropriate rank correlation coefficient. Kendall tau a is more likely than the Spearman correlation coefficient to prefer the true model over a simplified model that predicts tied ranks for a subset of pairs of dissimilarities (Nili et al., 2014). For each model, we computed the correlation coefficient between each participants’ judgment RDM and the RDMs predicted by the models. We first determined whether each of the model-prediction RDMs was significantly related to the similarity-judgment RDMs using a random-effects analysis across subjects (one-sided Wilcoxon signed-rank test). We corrected for multiple comparisons by controlling the expected false discovery rate at 0.05. We subsequently tested for differences in model performance. We performed pairwise model comparisons using a two-sided Wilcoxon signed-rank test. We corrected for multiple comparisons by controlling the expected false discovery rate at 0.05.

## Results

**FIGURE 3:**
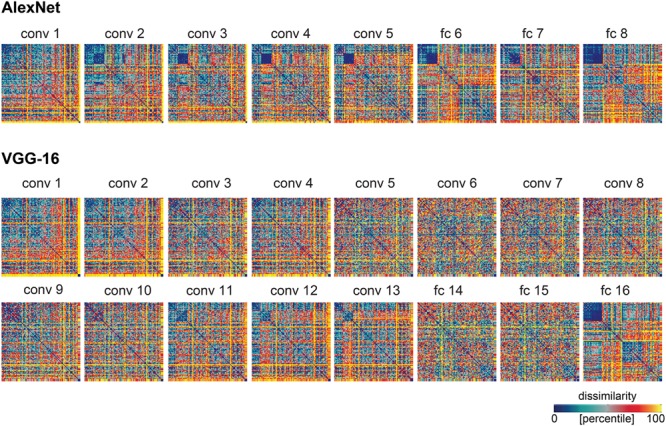
Model predictions of the similarity judgments for each DNN layer. RDMs for each layer of each network, after weighting the feature maps (convolutional layers) or model units (fully-connected layers) within that layer to best predict the human similarity judgments. Values within each RDM were transformed into percentiles for visualization (see color bar).

**FIGURE 4:**
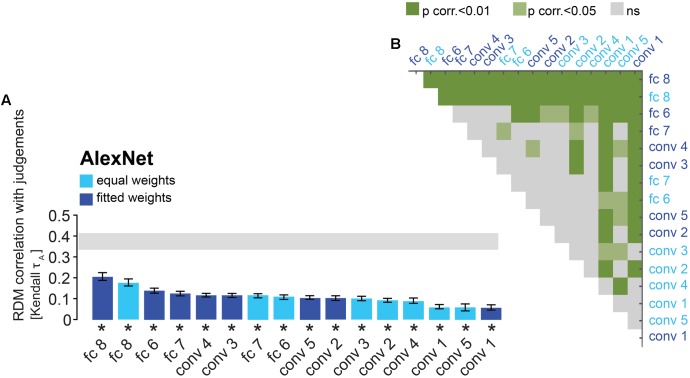
AlexNet performance at explaining similarity judgments: reweighting improves model performance for late layers. **(A)** Bars show the correlation between the similarity-judgment RDMs and each AlexNet layer RDM, with and without reweighting. A significant correlation between a layer RDM and the similarity-judgment RDMs is indicated by an asterisk (one-sided Wilcoxon signed-rank test, *p* < 0.05 corrected). Error bars show the standard error of the mean based on single-subject correlations, i.e., correlations between the single-subject similarity-judgment RDMs and a DNN RDM. The gray bar represents the noise ceiling, which indicates the expected performance of the true model given the noise in the data. “conv” indicates a convolutional layer and “fc” indicates a fully-connected layer. **(B)** Pairwise differences between model performance of AlexNet layer RDMs, with and without reweighting. Green color indicates significant pairwise differences (dark green *p* < 0.01, light green *p* < 0.05, FDR corrected across all comparisons).

**FIGURE 5:**
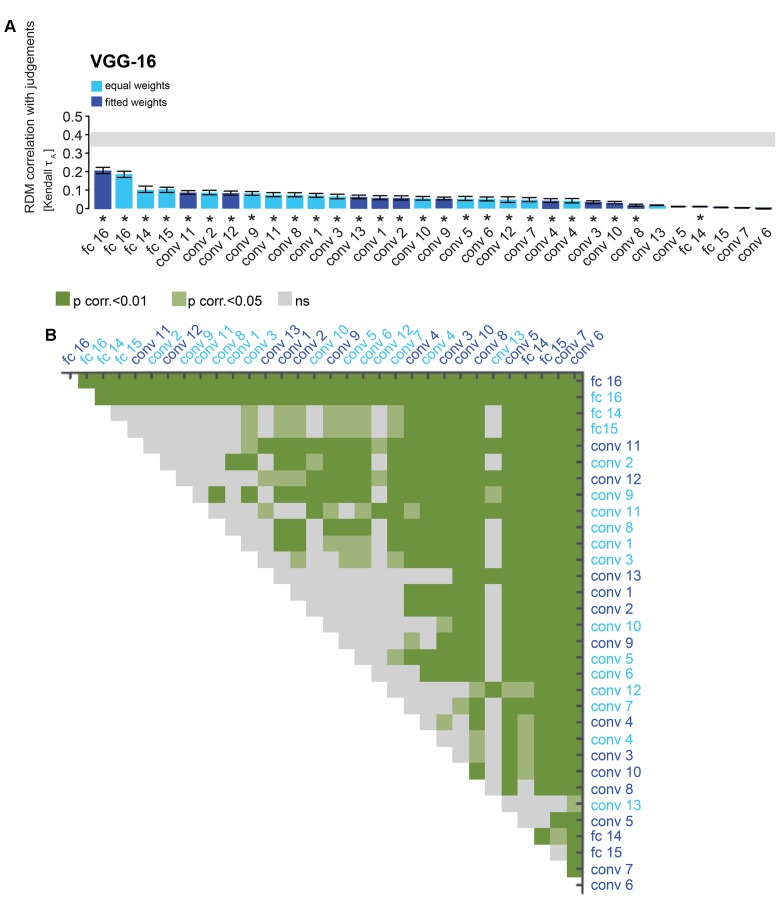
VGG-16 performance at explaining similarity judgments: reweighting improves model performance for layer 16 and late convolutional layers. **(A)** Bars show the correlation between the similarity-judgment RDMs and each VGG-16 layer RDM, with and without reweighting. Noise ceiling and significant correlations are indicated using the same conventions as in **Figure 4**. **(B)** Pairwise differences between model performance of VGG-16 layer RDMs, with and without reweighting, using the same conventions as in **Figure 4**.

**FIGURE 6:**
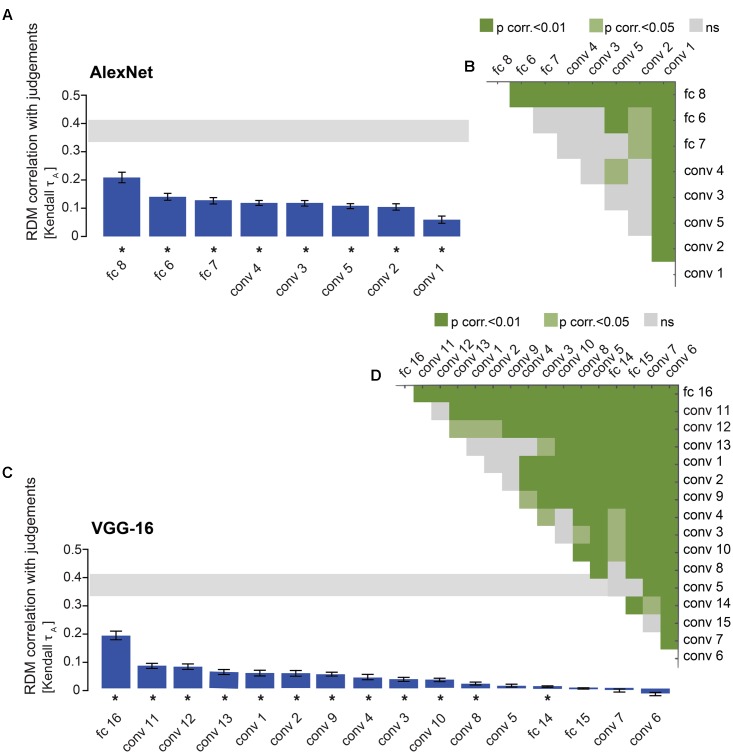
DNN performance at explaining similarity judgments: late layers outperform early layers. **(A)** Bars show the correlation between the similarity-judgment RDMs and each fitted AlexNet layer RDM. Noise ceiling and significant correlations are indicated using the same conventions as in **Figure 4**. **(B)** Pairwise differences between model performance of fitted AlexNet layer RDMs, using the same conventions as in **Figure 4**. **(C)** Bars show the correlation between the similarity-judgment RDMs and each fitted VGG-16 layer RDM. **(D)** Pairwise differences between model performance of fitted VGG-16 layer RDMs.

**FIGURE 7:**
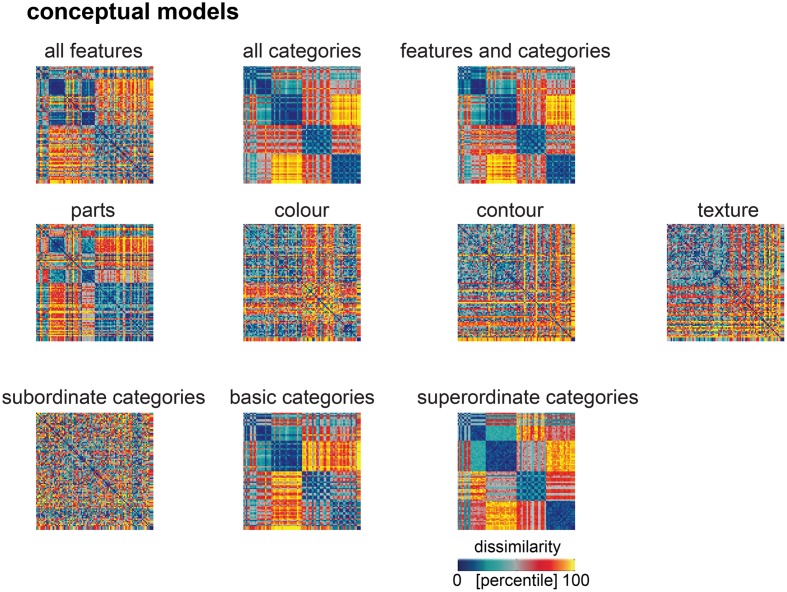
Model predictions of the similarity judgments for each conceptual model. RDMs for each conceptual model, after weighting the single-feature and single-category RDM models to best predict the human similarity judgments. We used a total of 10 conceptual models, derived from labels describing the 92 object images, which were generated by human observers. From the visual-feature descriptions, we constructed four feature-based RDMs, capturing object dissimilarity in terms of object parts, color, contour and texture. From the category labels, we constructed three separate categorical RDMs, capturing object dissimilarity in terms of subordinate, basic or superordinate categories. We also constructed comprehensive feature-based and categorical RDMs, by weighting all single-feature and all single-category RDMs, respectively, to best predict the similarity judgements, and a combined model, by weighting all single-feature and single-category RDMs models together to best predict the similarity judgements. Values within each RDM were transformed into percentiles for visualization (see color bar).

**FIGURE 8:**
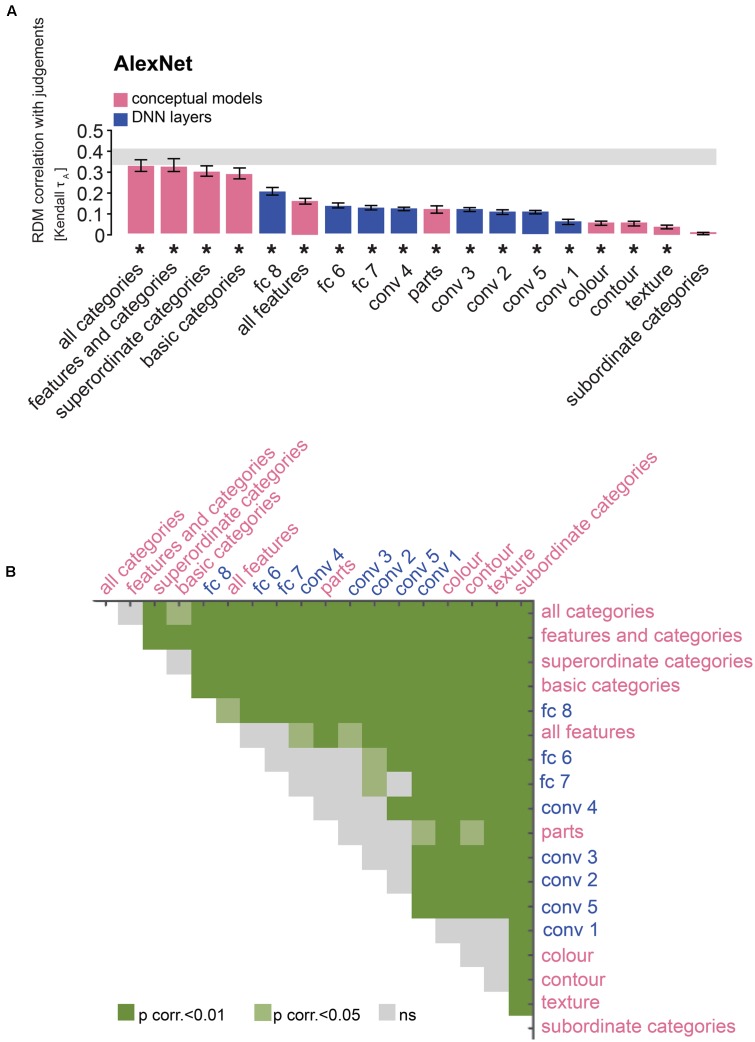
Comparison of AlexNet and conceptual models: the last layer outperforms feature-based, but not categorical models. **(A)** Bars show the correlation between the similarity-judgment RDMs and each fitted AlexNet layer RDM, as well as each fitted conceptual model. Noise ceiling and significant correlations are indicated using the conventions in **Figure 4**. **(B)** Pairwise differences between model performance of fitted AlexNet layer RDMs and conceptual models, using the same conventions as in **Figure 4**.

**FIGURE 9:**
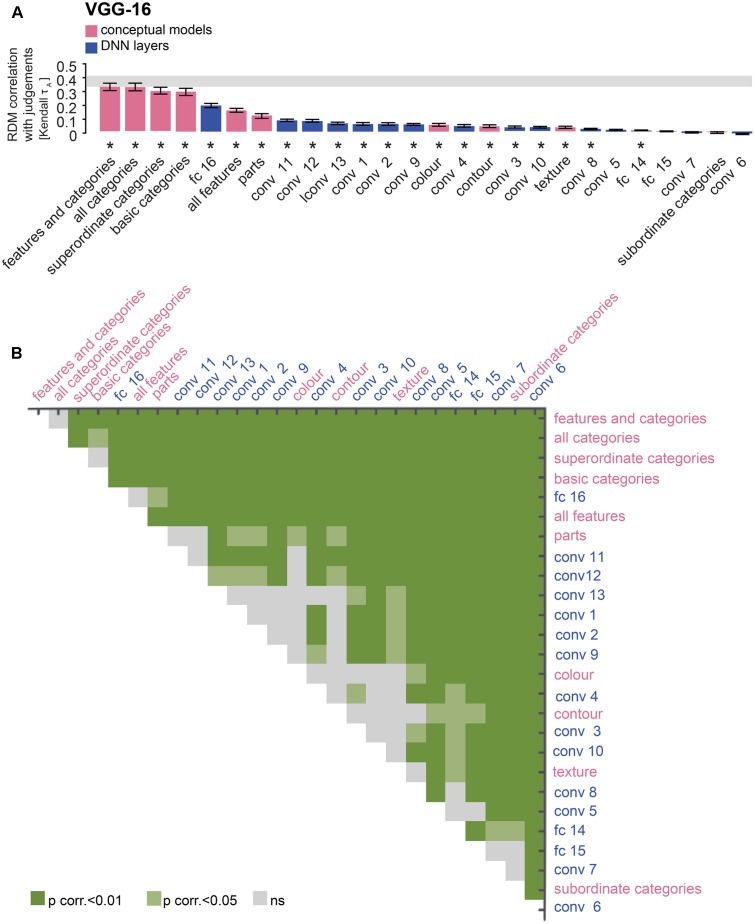
Comparison of VGG-16 and conceptual models: the last layer outperforms almost all feature-based, but not categorical models. **(A)** Bars show the correlation between the similarity-judgment RDMs and each fitted VGG-16 layer RDM, as well as each fitted conceptual model. Noise ceiling and significant correlations are indicated using the conventions in **Figure 4**. **(B)** Pairwise differences between model performance of fitted VGG-16 layer RDMs and conceptual models, using the same conventions as in **Figure 4**.

**FIGURE 10:**
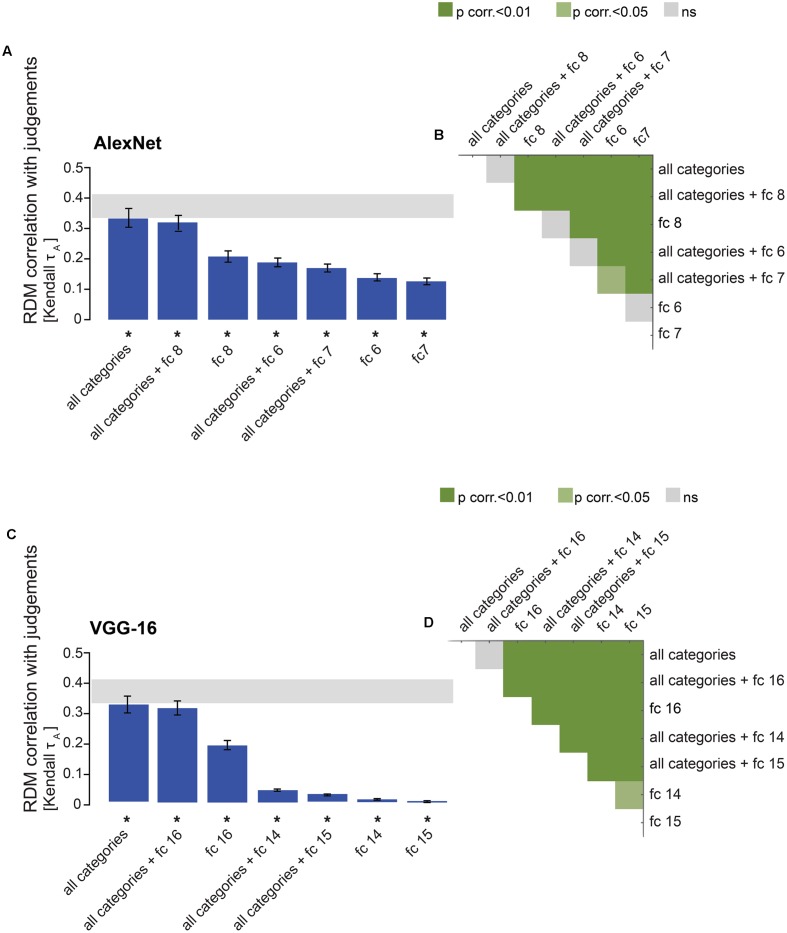
Combining categorical models with late DNN layers: no improvement in model performance. **(A)** Bars show the correlation between the similarity-judgment RDMs and predictions of the fitted “all categories” model, the fitted AlexNet fully-connected layers, and the fitted combined models. Noise ceiling and significant correlations are indicated using the conventions in **Figure 4**. **(B)** Pairwise differences between model performance of fully-connected AlexNet layer RDMs on their own or combined with the “all categories” model RDM, using the same conventions as in **Figure 4**. **(C)** Bars show the correlation between the similarity-judgment RDMs and predictions of the fitted “all categories” model, the fitted VGG-16 fully-connected layers, and the fitted combined models. Noise ceiling and significant correlations are indicated using the conventions in **Figure 4**. **(D)** Pairwise differences between model performance of fully-connected VGG-16 layer RDMs on their own, or combined with the “all categories” model RDM, using the same conventions as in **Figure 4**.

**FIGURE 11:**
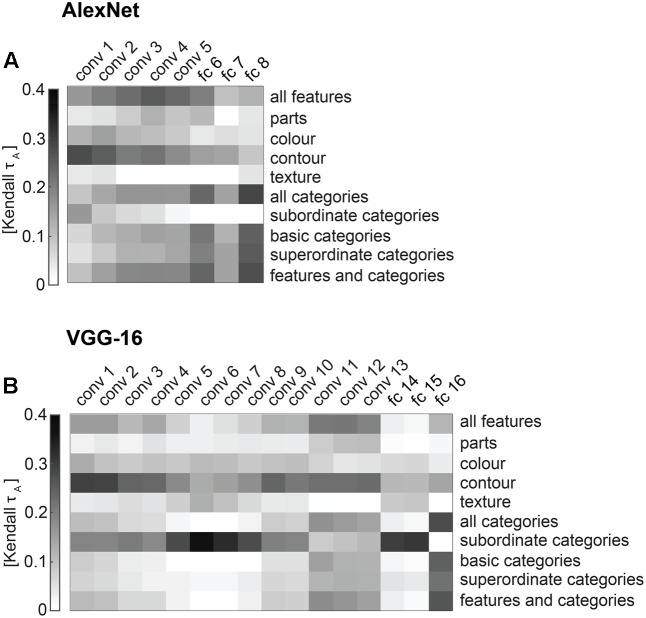
Correlations between DNN and conceptual model predictions of the similarity judgments. **(A)** The matrix shows pairwise correlations of AlexNet layer RDMs and conceptual models (Kendal tau a). **(B)** The matrix shows pairwise correlations of VGG-16 layer RDMs and conceptual models (Kendal tau a).

The human object-similarity judgments are displayed in **Figure 1**. The judgments show four main clusters corresponding to humans, non-human animals, natural objects, and manmade objects, and a tight cluster of human faces. We computed DNN representations of the same object images in AlexNet and VGG-16 (**Figure 2**), two DNNs that show high performance on object classification. These representations are shown in **Figure 3**. For convolutional layers, we linearly combined the layer’s feature-map representations, whereas for fully-connected layers we linearly combined individual model-unit representations to best predict the similarity judgments (**Figure 3** shows predictions for data held out during fitting). The DNN representations share several features with the similarity judgments, e.g., a tight cluster of human faces and a looser cluster of human faces and bodies. Representations in the last layer of AlexNet show the emergence of the four main clusters present in the similarity judgments. However, in the last layer of VGG-16, only the clusters of humans and natural objects can be observed. To investigate the extent to which different DNN layers explain similarity judgments, we correlated the layer-specific DNN representations with the similarity judgments and compared performance between layers (**Figures 4**–**6**). To investigate the relationship between DNNs and conceptual models in explaining similarity judgments, we implemented the following analyses. First, we compared DNN performance to the performance of conceptual models derived from feature-based and categorical object descriptions (**Figures 7**–**9**). Second, we combined the best performing conceptual model with late DNN layers to determine whether this improved model performance (**Figure 10**). Third, we correlated the conceptual model representations with DNN layer representations to directly examine commonalities between conceptual and image-computable representations (**Figure 11**).

### Representational Weighting Improves Performance of Late DNN Layers

Performance of DNN layers at explaining the similarity judgments might be improved by linearly reweighting the representations in each layer. To test this hypothesis, we implemented regularized (non-negative L2) linear regression to estimate the feature-map and model-unit weights that best predict the similarity-judgment RDM. The weights were estimated using a cross-validation procedure to prevent overfitting to the particular images used for fitting. The procedure is schematically represented in **Figure 2B**. We fitted feature maps for convolutional layers and model units for fully-connected layers. We assessed the performance of fitted (“fitted weights”) and unfitted (“equal weights”) layers at explaining the similarity judgments by correlating the DNN representations to the similarity judgments. The large majority of both fitted and unfitted layers significantly correlates with human similarity judgments (**Figures 4**, **5**; one-sided Wilcoxon signed-rank test; *p* < 0.05 corrected). Pairwise comparisons between layers show that linear reweighting improves performance for late convolutional layers and fully-connected layers across DNNs (two-sided Wilcoxon signed-rank test; *p* < 0.05 corrected), with the exception of VGG-16 layers 14 and 15. Performance of early and intermediate convolutional layers is not significantly affected or reduced by linear reweighting (**Figures 4**, **5**). None of the layers reach the noise ceiling, suggesting that the models can still be improved. We conclude that weighting of DNN feature maps and model units improves the performance of late layers in explaining similarity judgments. We therefore only include fitted layers for further analyses.

### Late DNN Layers Outperform Early DNN Layers in Explaining Similarity Judgments

The RDMs in **Figure 3** suggest that object representations in late layers might explain the similarity judgments better than early layers. To test this hypothesis, we compared model performance between layers. Results indicate that AlexNet layer 8 performs better than earlier AlexNet layers (layers 1–7) at explaining the similarity judgments (**Figures 6A,B**; two-sided Wilcoxon signed-rank test; *p* < 0.05 corrected). Furthermore, fully-connected layers 6 and 7 outperform some of the earlier, convolutional, layers, and among the convolutional layers, layers 2–5 perform better than layer 1. For VGG-16, layer 16 explains significantly more variance in the similarity judgments than earlier layers (**Figures 6C,D**; two-sided Wilcoxon signed-rank test; *p* < 0.05 corrected). Furthermore, late convolutional layers outperform most of the earlier convolutional layers. Because we see categorical, superordinate representations in similarity judgments, it seems plausible that late DNN layers with a higher level of abstraction and more categorical representations explain the behavioral data better. The extent to which AlexNet and VGG-16 explain similarity judgments appears very similar. It is important to note that VGG-16 outperforms AlexNet on image classification (29.5% top-1 error rate and 42.6% top-1 error rate, respectively) but is not better at explaining the similarity judgments. This suggests that the additional depth and superior classification performance of VGG-16 may not be advantageous in explaining human object similarity judgments.

### Late DNN Layers Outperform Most Feature-Based Models But Not Categorical Models

Human similarity judgments contain a strong categorical component. Consistent with this observation, we have previously shown that similarity judgments are better explained by the categorical than the feature-based model (Jozwik et al., 2016). Do late DNN layers, with a categorical structure created by their idiosyncratic training task, explain judgments as well as a categorical model derived from human classifications? To evaluate this, we compared performance at explaining the similarity judgments between DNN layers and feature-based and categorical models. **Figure 7** shows predictions for data held out during fitting for feature-based and categorical models. Results are shown in **Figures 8**, **9**. The full feature-based model is called “all features”. The full categorical model is called “all categories”. It combines all categories, forming a hierarchically nested set of category labels. We also weighted all feature and category predictors together in the “features and categories” model.

The large majority of DNN layers and almost all feature-based and categorical models, with the exception of the subordinate category model, significantly explain the similarity judgments. As expected, late AlexNet layer 8 outperforms all feature-based models (**Figures 8A,B**; two-sided Wilcoxon signed-rank test; *p* < 0.05 corrected). Other AlexNet layers outperform some feature-based models, i.e., the texture, contour and color models, but not others, i.e., the object-parts model and the “all features” model, which includes the object-parts model. In contrast, all layers, including the last layer, are outperformed by the categorical models, again with the exception of the subordinate category model. For VGG-16, late layer 16 outperforms all feature-based models except the “all features” model. Other VGG-16 layers in general do not outperform the feature-based models, except for a few convolutional layers that outperform the texture and contour models (**Figures 9A,B**; two-sided Wilcoxon signed-rank test; *p* < 0.05 corrected). As for AlexNet, all VGG-16 layers, including the last layer, are outperformed by the categorical models, again with the exception of the subordinate category model. These findings may indicate that DNNs represent object features well, but that their representation of basic and superordinate categories differs from those extracted by humans.

### Combining the Categorical Model with Late DNN Layers Does Not Improve Model Performance

Although the performance of the categorical models is very close to the noise ceiling, these models do not reach the ceiling. Perhaps the categorical models do not fully capture human shape sensitivity. Shape sensitivity, on the contrary, seems to be captured well by late DNN layers (Kubilius et al., 2016). Therefore, we combined the “all categories” model with each of the fully-connected DNN layers to test whether the noise ceiling can be reached by the combined model. We weighted the categorical model together with each of the fully-connected layers. We show that combining the categorical model with the fully-connected layers does not improve model performance (**Figure 10**). For AlexNet, the categorical model combined with layer 8 does not perform significantly differently from the categorical model on its own, but it performs significantly better than layer 8 (**Figures 10A,B**). The categorical model combined with layers 6 and 7 performs worse than the categorical model on its own, possibly due to the large number of parameters being fitted for the combined models, but better than layers 6 and 7 on their own. A similar pattern of results is observed for VGG-16. The categorical model combined with layer 16 does not perform significantly differently from the categorical model on its own, but it performs significantly better than layer 16 (**Figures 10C,D**). The categorical model combined with layer 14 or 15 performs worse than the categorical model on its own, but better than layers 14 and 15 on their own. These results suggest that, for the purpose of explaining object-similarity judgments, fully-connected layers do not seem to capture useful additional stimulus information that is not present in the categorical model.

### Correlations between Conceptual Models and DNN Layers Reveal a Progression from Feature-Based to Categorical Representations from Early to Late Layers

Comparing performance between DNN layers and conceptual models at explaining similarity judgments is informative. However, because similar model performance does not necessarily indicate that models explain the same variance, this approach does not directly address how similar stimulus representations are between the two types of models. Given the closer proximity of late DNN layers to the final category readout, we might expect the stimulus representation in late layers more closely match the stimulus representation in our categorical models. In contrast, representations within earlier layers do not show a clear categorical structure, and might be driven more strongly by (lower-level) visual features, and may correlate better with representations in feature-based models. To investigate this, we correlated every conceptual model with every DNN layer (**Figures 11A,B**). In both networks (earlier) convolutional layers indeed appear to be more strongly correlated with feature-based than categorical models, while fully-connected layers show the opposite pattern.

In addition to this general observation, we highlight a few additional more detailed observations below. First, among convolutional layers, the predominance of feature-based over categorical information seems to diminish with increasing depth, further supporting the observed progression from feature-based to categorical representations from early to late layers. Second, among all layers, feature-based models based on object parts (“all features” and parts) appear to correlate best with late convolutional layers, suggesting that these layers might represent object features of intermediate complexity that are relevant for object categorisation. Third, although contour information is not very useful for explaining similarity judgments (**Figures 8A**, **9A**), it appears to be represented consistently across convolutional layers, as indicated by moderate positive correlations between the contour model and convolutional layers in both networks (**Figure 11**). Fourth, the subordinate category model, which cannot explain the similarity judgments (**Figures 8A**, **9A**), does not correlate highly with any AlexNet layer, but does correlate moderately with intermediate convolutional layers and fully-connected layers 14 and 15 in VGG-16. Interestingly, these VGG-16 layers also cannot explain the similarity judgments well (**Figure 9A**), and in addition do not correlate highly with the other categorical models (**Figure 11B**). What does this mean? One potentially relevant characteristic shared by the subordinate-category model and the abovementioned VGG-16 layers, is that their representations are relatively sparse, predicting large dissimilarities for only a small number of image pairs (e.g., large dissimilarities for one specific image with all other images). Weights estimated for these representations might not generalize well to images held out during fitting, leading to low performance at explaining the similarity judgments. Furthermore, assuming overlap between specific subordinate-category predictors and VGG feature-map or model-unit predictors, and given identical cross-validation folds across models, the two types of models might yield similar predictions, which would be consistent with the moderate correlations observed in **Figure 11B**.

The pattern of correlations we observe between the conceptual models and DNN layers reveals the relationship between successive image processing steps performed by DNNs, and psychologically salient object properties such as features and categories. Early and intermediate layers correlate mostly with features, whereas late layers correlate mostly with categories. The pattern we observe follows the progression of information processing in the visual system, where features are represented in early visual cortex and categories in high-level visual cortex.

In summary, our results suggest that DNNs are reasonably good at modeling human object-similarity judgments, however they still need to be improved to fully explain the judgments. This applies particularly to the higher-level categorical component of human judgments, which might be difficult to infer from visual features alone. Given that the DNNs used in this study were not trained to judge object similarities, their performance at explaining the similarity judgments is quite impressive.

## Discussion

Deep convolutional Neural Networks have revolutionized computer vision in recent years, reaching human-level performance on object classification (Krizhevsky et al., 2012; Simonyan and Zisserman, 2015; He et al., 2016). DNNs perform considerably well at predicting object representations across the primate visual system (Khaligh-Razavi and Kriegeskorte, 2014; Yamins et al., 2014; Güçlü and van Gerven, 2015; Cichy et al., 2016; Hong et al., 2016), and might be able to predict human behavior in cognitive tasks based on visual object representations. Indeed, DNNs have recently proven successful at predicting judgments of category typicality (Lake et al., 2015), object memorability (Dubey et al., 2015), and object similarity (Kubilius et al., 2016; Peterson et al., 2016), especially after linearly reweighting model representations to best predict human behavior (Peterson et al., 2016). Here, we show that two DNNs of different depths (AlexNet and VGG-16) significantly explain human similarity judgments for real-world object images from a wide range of natural categories, replicating and extending previous findings. Furthermore, to better understand what stimulus information DNNs represent, we compared their performance at explaining similarity judgments, as well as their internal representations, to those of conceptual models derived from human perception. The conceptual models were based on visual-feature and category descriptions of the object images, generated by human observers (Jozwik et al., 2016). We show that the last fully-connected DNN layers outperform most feature-based models, but not categorical models. Our findings suggest that further work may be needed to bring high-level semantic representations in DNNs closer to semantic representations extracted by humans.

### Weighted Representational Modeling Improves Performance of Late DNN Layers at Explaining Similarity Judgments

Weighted representational modeling previously proved successful in increasing the performance of both conceptual (Jozwik et al., 2016) and DNN models (Khaligh-Razavi and Kriegeskorte, 2014) at explaining object representations in brain and behavior. We applied the same approach here, using regularized (non-negative L2) linear regression to weight object representations in DNN layers to best explain human similarity judgments for real-world object images from a wide range of categories. We weighted feature maps for convolutional layers and model units for fully-connected layers. We show an increase in performance for late convolutional and fully-connected DNN layers after weighting. A similar approach to increase the power of DNNs to explain human behavior through model fitting was described in Peterson et al. (2016). The authors fitted model units of penultimate DNN layers using regularized (L2) linear regression, yielding an increase in performance at explaining human object-similarity judgments for a range of DNNs, including CaffeNet (AlexNet) and VGG-16, consistent with our findings. Increases in model performance for the current study appear less impressive than those reported by Peterson et al. (2016), possibly because of a difference in the cross-validation procedure used to generate model predictions. Peterson et al. (2016) obtain model predictions by cross validating to similarity judgments (not images) held out during fitting, leaving open whether the fitted models generalize well to new images. Indeed, when they use their VGG-16 model to classify animals in images held out during fitting, the fitted model performs consistently worse than the unfitted model (Peterson et al., 2016). Nevertheless, model performance is still very good, suggesting that other factors might play a role as well. These factors include image background and range of categories tested. Preliminary observations indicate that DNN representations of isolated object images are sparser than those of object images with natural backgrounds, especially when the representations are standardized before fitting, as in the current study. Sparser representations might lead to lower generalization performance to new images. In addition, the wider range of categories tested in the current study may invite high-level, abstract categories in the similarity judgments, which might be more challenging to explain with DNNs not explicitly trained to distinguish these categories. Another difference in results is that Peterson et al. (2016) found that VGG-16 performed better than AlexNet, which is not the case in our study. It is possible that we did not see the advantage of the deeper architecture because we used isolated objects instead of objects displayed on a natural background. In contrast to VGG-16, AlexNet retains more unrelated background information in the last convolutional layer, which disturbs the final prediction if background information is present (Yu et al., 2016). It is possible that we would observe an advantage of VGG-16 over AlexNet in explaining similarity judgments of images with a natural background. We deliberately did not include other DNNs in our study, as we wanted to focus on networks with similar architectures but different depths, and these criteria are fulfilled by VGG-16 and AlexNet. More recent state-of-the-art networks such as GoogleNet (Szegedy et al., 2013) or deep residual networks (He et al., 2016) have substantial architectural differences, including nested convolutions and skipping connections, respectively.

In future studies, the size of the network might be reduced by keeping only the feature maps or model units that are consistently assigned high weights during the fitting procedure. Model dimensions that consistently receive low weights during fitting might not significantly contribute to explaining human neural or cognitive representations and may be eliminated to create a more succinct model of human cognition. It would be interesting to evaluate whether the feature maps and model units that contribute most to explaining human brain and behavior also have greater importance within DNNs when classifying objects. More broadly, DNNs built and trained for engineering purposes provide an excellent starting point for modeling human perception and cognition, and there are many further opportunities to tailor and interpret such networks.

### Late DNN Layers Outperform Early DNN Layers in Explaining Similarity Judgments

We show that late DNN layers outperform early DNN layers in explaining similarity judgments. As categorical, high-level object properties are reflected in similarity judgments, it is intuitive that late DNN layers with a higher level of abstraction and more complex representations explain behavioral data better. Our finding is consistent with previous studies where later layers performed better than early layers at explaining shape sensitivity and similarity judgments of real-world object images (Kubilius et al., 2016; Peterson et al., 2016). Our findings replicate and extend these previous findings to a rich stimulus set consisting of colored real-world object images from a wide range of categories, including animate and inanimate objects.

We found that two DNNs with similar architectures but different depths and object classification accuracies yielded qualitatively similar conclusions. A network with a deeper architecture (VGG-16) did not perform better than a network with a less deep architecture (AlexNet). As discussed in the previous paragraph, this finding differs from a previous study, where a network with a deeper architecture (VGG-16) was reported to perform better than AlexNet at explaining similarity judgments of animal images (Peterson et al., 2016). These varied results might be due to any of multiple differences between our study and Peterson et al. (2016), including differences in image background (uniform gray versus natural), the range of categories tested, and the similarity-judgment task. Any differences in the information transformations within the tested DNNs of different depths appear unimportant for explaining similarity judgments of isolated real-world objects. Although a push toward wider (i.e., a larger number of feature maps in each layer) and deeper (i.e., more layers) architectures over the past 5 years has increased the performance of DNNs on object classification tasks, bigger networks might not necessarily predict human behavior better than smaller ones. The neuroscience and psychology communities may establish alternative benchmarks for future DNN development, which involve explaining aspects of human brain and cognition, to complement engineering benchmarks focused on task accuracy.

### DNNs Outperform Most Feature-Based Models, But Not Categorical Models, in Explaining Similarity Judgments

We compared the DNNs’ performance to that of conceptual models derived from feature-based and categorical object descriptions. We demonstrate that late DNN layers outperform most feature-based models, but not categorical models. This finding is consistent with a previous finding that DNNs explain more variance in shape judgments than category judgments (Kubilius et al., 2016). Moreover, AlexNet explained inferior temporal cortex representations of images to a high extent only when features were remixed to emphasize an animate-versus-inanimate object distinction (Khaligh-Razavi and Kriegeskorte, 2014). This suggests that DNNs may need additional training to arrive at similar categorical representations as humans, perhaps suggesting that the 1,000 object categories such networks are conventionally trained to detect (Russakovsky et al., 2015) are not well-matched in type or saliency to those extracted by humans. The resemblance between image properties represented by DNNs and those extracted by humans might be stronger for (lower-level) visual image features. This idea is consistent with visualizations of feature selectivities in intermediate and early DNN layers (Zeiler and Fergus, 2013; Yu et al., 2016). As revealed by such visualizations, layer 1 of a network similar in architecture to AlexNet appears to represent mostly colors and simple edges, which is consistent with our finding that the performance of layer 1 was similar to that of the color model. Layer 2 appears to represent slightly more complex features such as textures, and layers 3–5 appear to represent more complex features such as object parts and whole objects. The feature selectivities observed for layers 2–5 seem consistent with our finding that layers 2–5 perform similarly to the object-part model. The progressively higher match to human perceived object similarity corresponds to the progression within DNNs from local features to the emergence of whole objects that are finally assigned category labels.

We have previously shown that the object representation in inferior temporal (IT) cortex cannot fully explain the similarity judgments either (Mur et al., 2013). Both DNNs and IT therefore seem to be missing information about the stimuli that is captured by the categorical descriptions (which explain the object similarity judgments better than the DNNs and IT). These findings not only suggest that there is still room for improvement, but also suggest a direction for improvement, for example, training DNNs to classify objects into categories that are highly relevant to humans, such as faces and animals. It has been shown that features learned by deep nets are sufficient to account for object-level categorisation performance even for unseen categories (Rajalingham et al., 2015). However, it remains to be tested whether a more natural image training set would yield a better prediction for a range of unseen categories.

### What Stimulus Information Do Different DNN Layers Represent?

Deep convolutional Neural Networks seem to be missing stimulus information for explaining the similarity judgments that is captured by the categorical models. Indeed, combining the categorical model with the fully-connected layers improves their performance. Is the reverse true as well? Do DNNs capture stimulus information for explaining similarity judgments that the categorical models are missing? Our results indicate that this is not the case: the combined models do not perform better than the categorical model on its own. These findings suggest that any categorical information for explaining similarity judgments that is represented by the late DNN layers is also captured by the categorical model. We further investigated the relationship between DNN representations and conceptual models by correlating their model predictions. Results show a progression from feature-based to categorical representations from early to late layers. Contour and color seem to be represented most strongly by early convolutional layers, and object parts by late convolutional layers. Basic and superordinate categories appear to be most strongly represented by fully-connected layers, especially by the last scores layer. These results are roughly consistent with the stages of visual processing along the ventral visual stream: representations show selectivity to increasingly complex visual features with increasing depth. This result is further consistent with findings that representations in early DNN layers correlate most strongly with representations in early visual cortex and representations in late DNN layers correlate most strongly with representations in high-level visual cortex (Khaligh-Razavi and Kriegeskorte, 2014; Güçlü and van Gerven, 2015; Cichy et al., 2016; Hong et al., 2016; Yamins and DiCarlo, 2016).

### DNNs as Potential Models for Explaining Human Cognition

Late layers of DNNs trained on image categorisation explained human object-similarity judgments to a considerable extent despite not being trained explicitly to make similarity judgments. However, the DNNs did not explain all explainable variance in the similarity judgments, leaving scope for changes to the training, visual diet and/or architecture of the DNNs to fully explain human behavior on this task. For example, because human perception involves recurrent as well as feedforward processing, recurrent DNN models may better predict human behavior on similarity-judgment tasks.

It is important not to equate DNNs’ performance on categorisation tasks with their ability to explain human cognition. There may be differences in the computational problems solved by DNNs and humans. Further work is needed to modify DNNs using approaches that are inspired by human behavior, e.g., using only feature maps in the network that received non-zero weights when fitting DNNs layers to similarity judgments, as presented in this study, or altering training tasks to include and emphasize human-salient categories such as those contained within our categorical model.

Still, the fact that DNNs not trained on a similarity judgment task explained human behavior in such a task points to the flexibility of the representations learned by DNNs. The current architecture and training of DNNs already makes them somewhat able to generalize across tasks, as shown in the current study. Further development in engineering will lead to DNNs that can perform multiple tasks. Such general DNNs could be very promising for explaining brain representations that must flexibly support many different tasks.

Exploring the relationship between DNNs and human perception cannot only help us understand human cognition better but may also inform artificial intelligence approaches to build better DNN models. For many applications, we want artificial systems that are sensitive to dimensions that are perceptually and cognitively relevant to us.

## Conclusion

Similarity within the last DNN layers predicted human perceived object similarity better than feature-based models capturing object parts, colors, textures, and contours, but not as well as models capturing basic and superordinate categories. Linearly reweighting feature maps in late convolutional layers and model units in fully-connected layers proves to be useful for increasing the explanatory power of these layers. For the networks tested here with isolated real-world object images, increased depth was not associated with increased ability to explain similarity judgments. By comparing DNN representations to simpler conceptual models we gain insight into aspects of the DNNs’ representations that contribute to their explanatory power, and into those that are missing. In summary, DNNs explain human similarity judgments impressively despite not being trained on this specific cognitive task, and have potential to explain many aspects of human cognition.

## Ethics Statement

This study was carried out in accordance with the recommendations of the Cambridge Psychology Research Ethics Committee with written informed consent from all subjects. All subjects gave written informed consent in accordance with the Declaration of Helsinki. The protocol was approved by the Cambridge Psychology Research Ethics Committee.

## Author Contributions

KMJ and MM performed experiments. KMJ and MM analyzed the data. KRS provided DNN representations. KMJ, KRS, and MM wrote the manuscript. MM and NK provided conceptual guidance.

## Conflict of Interest Statement

The authors declare that the research was conducted in the absence of any commercial or financial relationships that could be construed as a potential conflict of interest.
