# Supplementary material for: Deep Convolutional Neural Networks Outperform Feature-Based But Not Categorical Models in Explaining Object Similarity Judgments
Source: Front Psychol. 2017 Oct 9;8:1726. doi: 10.3389/fpsyg.2017.01726 (PMC5640771; doi:10.3389/fpsyg.2017.01726)
Supplement: Supplementary file 1 [file Table_1.pdf]

**Supplementary Table 1. List of predictors for the conceptual models.**

| | all features | parts | colour | contour | texture | all categories | subordinate categories | basic categories | superordinate categories |
|---|---|---|---|---|---|---|---|---|---|
| 1 | arched | arm | black | arched | brick | animal | armadillo | animal | artificial |
| 2 | arm | back | blonde | arrow | furry | armadillo | aubergine | body part | natural |
| 3 | arrow | beard | blue | coiled | glass | artificial | baboon | building | food/edible |
| 4 | back | branches | brown | curved | hairy | aubergine | banana | carnivore | organism/living |
| 5 | beard | brick | green | cylindrical | leafy | baboon | bottle | cold-blooded | nonliving/manmade |
| 6 | black | bristles | grey | domed | long | banana | building | entrance | |
| 7 | blonde | building | red | pear-shaped | metallic | body part | camel | face | |
| 8 | blue | cheeks | white | rectangular | plastic | bottle | carrots | fruit | |
| 9 | branches | chest | wooden | rounded | shiny | building | chimpanzee | gesture | |
| 10 | brick | collar | woolly | spiky | spiky | camel | courgette | hand | |
| 11 | bristles | core | yellow | straight | stubbly | carnivore | crocodile | herbivore | |
| 12 | brown | dimples | socks/pink | symmetrical | sharp/scaly | carrots | dancer | home | |
| 13 | building | dress | wet/water | cloves/bulbous | spire/ steeple/tall | chimpanzee | door | horned | |
| 14 | cheeks | ear | purple/seat/wheels | round/circular | beak/feathers/feathery/wings | cold-blooded | elephant | landscape | |
| 15 | chest | eye | | cubic/hob/square | | courgette | fist | limb | |
| 16 | coiled | eyelashes | | | | crocodile | garlic | livestock | |
| 17 | collar | feet | | | | dancer | giraffe | male | |
| 18 | core | fur | | | | door | goat | mammal | |
| 19 | curved | glasses | | | | elephant | grapes | man | |
| 20 | cylindrical | goatee | | | | entrance | great dane | monkey | |
| 21 | dimples | hair | | | | face | hammer | object | |
| 22 | domed | handle | | | | fist | hand | primate | |
| 23 | dress | head | | | | fruit | key | reptile | |
| 24 | ear | hooves | | | | garlic | kiwi | road | |
| 25 | eye | horns | | | | gesture | lettuce | salad | |
| 26 | eyelashes | humps | | | | giraffe | man | sense | |
| 27 | feet | knuckles | | | | goat | monkey | shelter | |
| 28 | flesh | leaves | | | | grapes | pear | symbol | |
| 29 | fur | lips | | | | great dane | pepper | technology | |
| 30 | furry | moustache | | | | hammer | pinecone | tree | |
| 31 | glass | nail | | | | hand | pineapple | urban | |
| 32 | glasses | neck | | | | herbivore | radish | vegetable | |
| 33 | goatee | necklace | | | | home | red pepper | vertebrate | |
| 34 | green | nostrils | | | | horned | roundabout sign | warning | |
| 35 | grey | overalls | | | | key | sheep | frog/amphibian | |
| 36 | hair | palm | | | | kiwi | snake | structure/architecture | |
| 37 | hairy | plants | | | | landscape | stop sign | potato/carbohydrate | |
| 38 | handle | pole | | | | lettuce | tomato | occupation/chef | |
| 39 | head | roof | | | | limb | toothbrush | spice/chili | |
| 40 | hooves | seeds | | | | livestock | tree | religion/church | |
| 41 | horns | shadow | | | | male | umbrella | skyscrapers/city | |
| 42 | humps | shirt | | | | mammal | waterfall | roof/dome | |
| 43 | knuckles | shoes | | | | man | zebra | house/dwelling | |
| 44 | leafy | shoulder | | | | monkey | frog/amphibian | hearing/ear | |
| 45 | leaves | skin | | | | natural | potato/carbohydrate | food/edible | |
| 46 | lips | sky | | | | object | occupation/chef | tool/equipment | |
| 47 | long | snout | | | | pear | spice/chili | woman/female | |
| 48 | metallic | soil | | | | pepper | religion/church | weapon/gun | |
| 49 | moustache | stem | | | | pinecone | skyscrapers/city | pliers/implement | |
| 50 | nail | step | | | | pineapple | roof/dome | water/lake | |
| 51 | neck | stripes | | | | primate | house/dwelling | maple leaf/leaf | |
| 52 | necklace | stubble | | | | radish | hearing/ear | organism/living | |
| 53 | nostrils | sunglasses | | | | red pepper | woman/female | wood/logs | |
| 54 | overalls | tail | | | | reptile | wolf/fox | sign/road sign | |
| 55 | palm | tape | | | | road | weapon/gun | adult/human/person | |
| 56 | pear-shaped | teeth | | | | roundabout sign | wig/hair | apple/apple core/eaten | |
| 57 | plants | thumb | | | | salad | pliers/implement | arch/archway/monument | |
| 58 | plastic | tongue | | | | sense | water/lake | bird/flightless/ostrich | |
| 59 | pole | torso | | | | sheep | maple leaf/leaf | bovine/cattle/cow | |
| 60 | rectangular | tower | | | | shelter | wood/logs | boy/child/young | |
| 61 | red | tree | | | | snake | sign/road sign | bulb/light/light bulb | |
| 62 | roof | trigger | | | | stop sign | apple/apple core/eaten | canine/dog/pet | |
| 63 | rounded | waterfall | | | | symbol | arch/archway/monument | cassette tape/music/recording | |
| 64 | seeds | window | | | | technology | bird/flightless/ostrich | communication/mobile phone/phone | |
| 65 | shadow | wrist | | | | tomato | bovine/cattle/cow | country/flag/Japanese flag | |
| 66 | shiny | legs/body | | | | toothbrush | boy/child/young | knitting/wool/yarn | |
| 67 | shirt | cloves/bulbous | | | | tree | bulb/light/light bulb | big cat/cat/feline/lion | |
| 68 | shoes | wall/door | | | | umbrella | canine/dog/pet | chair/furniture/office chair/seat | |

| | | | | | | | | | |
|---|---|---|---|---|---|---|---|---|---|
| 69 | shoulder | forehead/eyebrow | | | | urban | cassette tape/music/recording | eye/organ/sight/vision | |
| 70 | skin | hand/fingers | | | | vegetable | communication/mobile phone/phone | bush/garden/plant/shrub/topiary | |
| 71 | sky | ground/grass | | | | vertebrate | country/flag/Japanese flag | appliance/cooker/cooking/kitchen/oven/stove | |
| 72 | snout | trousers/hat | | | | warning | knitting/wool/yarn | | |
| 73 | soil | path/house | | | | waterfall | big cat/cat/feline/lion | | |
| 74 | spiky | pupil/iris | | | | zebra | chair/furniture/office chair/seat | | |
| 75 | stem | whiskers/mane | | | | frog/amphibian | eye/organ/sight/vision | | |
| 76 | step | nose/mouth | | | | structure/architecture | bush/garden/plant/shrub/topiary | | |
| 77 | straight | socks/pink | | | | potato/carbohydrate | appliance/cooker/cooking/kitchen/oven/stove | | |
| 78 | stripes | tusks/trunk | | | | occupation/chef | | | |
| 79 | stubble | wet/water | | | | spice/chili | | | |
| 80 | stubbly | antenna/buttons/screen | | | | religion/church | | | |
| 81 | sunglasses | clouds/forest/lake | | | | skyscrapers/city | | | |
| 82 | symmetrical | cubic/hob/square | | | | roof/dome | | | |
| 83 | tail | purple/seat/wheels | | | | house/dwelling | | | |
| 84 | tape | spire/ steeple/tall | | | | hearing/ear | | | |
| 85 | teeth | beak/feathers/feathery/wings | | | | food/edible | | | |
| 86 | thumb | | | | | tool/equipment | | | |
| 87 | tongue | | | | | woman/female | | | |
| 88 | torso | | | | | wolf/fox | | | |
| 89 | tower | | | | | weapon/gun | | | |
| 90 | tree | | | | | wig/hair | | | |
| 91 | trigger | | | | | pliers/implement | | | |
| 92 | waterfall | | | | | water/lake | | | |
| 93 | white | | | | | maple leaf/leaf | | | |
| 94 | window | | | | | organism/living | | | |
| 95 | wooden | | | | | wood/logs | | | |
| 96 | woolly | | | | | nonliving/manmade | | | |
| 97 | wrist | | | | | sign/road sign | | | |
| 98 | yellow | | | | | adult/human/person | | | |
| 99 | legs/body | | | | | apple/apple core/eaten | | | |
| 100 | cloves/bulbous | | | | | arch/archway/monument | | | |
| 101 | round/circular | | | | | bird/flightless/ostrich | | | |
| 102 | wall/door | | | | | bovine/cattle/cow | | | |
| 103 | forehead/eyebrow | | | | | boy/child/young | | | |
| 104 | hand/fingers | | | | | bulb/light/light bulb | | | |
| 105 | ground/grass | | | | | canine/dog/pet | | | |
| 106 | trousers/hat | | | | | cassette tape/music/recording | | | |
| 107 | path/house | | | | | communication/mobile phone/phone | | | |
| 108 | pupil/iris | | | | | country/flag/Japanese flag | | | |
| 109 | whiskers/mane | | | | | knitting/wool/yarn | | | |
| 110 | nose/mouth | | | | | big cat/cat/feline/lion | | | |
| 111 | socks/pink | | | | | chair/furniture/office chair/seat | | | |
| 112 | sharp/scaly | | | | | eye/organ/sight/vision | | | |
| 113 | tusks/trunk | | | | | bush/garden/plant/shrub/topiary | | | |
| 114 | wet/water | | | | | appliance/cooker/cooking/kitchen/oven/stove | | | |
| 115 | antenna/buttons/screen | | | | | | | | |
| 116 | clouds/forest/lake | | | | | | | | |
| 117 | cubic/hob/square | | | | | | | | |
| 118 | purple/seat/wheels | | | | | | | | |
| 119 | spire/ steeple/tall | | | | | | | | |
| 120 | beak/feathers/feathery/wings | | | | | | | | |
